# Economic burden of COVID-19, China, January–March, 2020: a cost-of-illness study

**DOI:** 10.2471/BLT.20.267112

**Published:** 2020-11-30

**Authors:** Huajie Jin, Haiyin Wang, Xiao Li, Weiwei Zheng, Shanke Ye, Sheng Zhang, Jiahui Zhou, Mark Pennington

**Affiliations:** a King’s Health Economics, Institute of Psychiatry, Psychology & Neuroscience at King’s College London, Box 024, The David Goldberg Centre, London, SE5 8AF, England.; b Health Technology Assessment Research Department, Shanghai Health Development Research Centre, Shanghai, China.; c Centre for Health Economics Research and Modelling Infectious Diseases, University of Antwerp, Antwerp, Belgium.; d School of Public Health, Fudan University, Shanghai, China.; e Department of Infectious Disease, Shanghai Public Health Clinical Center, Shanghai, China.; f Tongji Medical College, Huazhong University of Science and Technology, Wuhan, China.; g School of Population and Global Health, The University of Western Australia, Perth, Australia.

## Abstract

**Objective:**

To estimate the economic cost of coronavirus disease 19 (COVID-19) in 31 provincial-level administrative regions and in total, in China.

**Methods:**

We used data from government reports, clinical guidelines and other publications to estimate the main cost components of COVID-19 during 1 January–31 March 2020. These components were: identification and diagnosis of close contacts; suspected cases and confirmed cases of COVID-19; treatment of COVID-19 cases; compulsory quarantine of close contacts and suspected cases; and productivity losses for all affected residents. Primary outcomes were total health-care and societal costs.

**Findings:**

The total estimated health-care and societal costs associated with COVID-19 were 4.26 billion Chinese yuan (¥; 0.62 billion United States dollars, US$) and ¥ 2646.70 billion (US$ 383.02 billion), respectively. Inpatient care accounted for 44.2% (¥ 0.95 billion/¥ 2.15 billion) of routine health-care costs followed by medicines, accounting for 32.5% (¥ 0.70 billion/¥ 2.15 billion). Productivity losses accounted for 99.8% (¥ 2641.61 billion/¥ 2646.70 billion) of societal costs, which were mostly attributable to the effect of movement-restriction policies on people who did not have COVID-19. Societal costs were most sensitive to salary costs and number of working days lost due to movement-restriction policies. Hubei province had the highest health-care cost while Guangdong province had the highest societal cost.

**Conclusion:**

Our results highlight the high economic burden of the COVID-19 outbreak in China. The control measures to prevent the spread of disease resulted in substantial costs from productivity losses amounting to 2.7% (US$ 382.29 billion/US$ 14.14 trillion) of China’s annual gross domestic product.

## Résumé

**Objectif:**

Estimer l'impact économique de la maladie à coronavirus 2019 (COVID-19) dans 31 régions administratives de niveau provincial ainsi que dans l'ensemble de la Chine.

**Méthodes:**

Nous avons utilisé les données issues des rapports officiels, des directives cliniques et d'autres publications pour évaluer les principales composantes de coût de la COVID-19 durant la période comprise entre le 1^er^ janvier et le 31 mars 2020. Ces composantes étaient les suivantes: identification et diagnostic des contacts rapprochés; cas suspects et confirmés de COVID-19; traitement des cas de COVID-19; quarantaine obligatoire des contacts rapprochés et cas suspects; et enfin, perte de productivité pour toutes les personnes concernées. Le coût total des soins de santé et le coût total pour la société constituaient les résultats primaires.

**Résultats:**

Nous avons estimé que le coût total des soins de santé et le coût total pour la société induits par la COVID-19 s'élevaient à 4,26 milliards de yuans (0,62 milliard de dollars américains) dans le premier cas et à 2646,70 milliards de yuans (383,02 milliards de dollars américains) dans le second. L'hospitalisation des patients a représenté 44,2% (0,95 milliard de yuans/2,15 milliards de yuans) des frais de soins de santé courants, suivie par les médicaments avec 32,5% (0,70 milliard de yuans/2,15 milliards de yuans). La perte de productivité a représenté 99,8% (2641,61 milliards de yuans/2646,70 milliards de yuans) du coût pour la société, principalement lié à l'impact des politiques de restriction des déplacements sur les personnes ne souffrant pas de la COVID-19. Le coût pour la société dépendait majoritairement des dépenses salariales et du nombre de jours de travail perdus à cause des politiques de restriction des déplacements. C'est la province du Hubei qui a déboursé le plus pour les soins de santé, tandis que celle du Guangdong a été la plus durement touchée en termes de coût pour la société.

**Conclusion:**

Nos résultats mettent en lumière le lourd fardeau économique de l'épidémie de COVID-19 en Chine. Les mesures de lutte contre la propagation du virus ont entraîné des pertes considérables en raison d'une baisse de productivité équivalant à 2,7% (382,29 milliards de dollars américains/14,14 billions de dollars américains) du produit intérieur brut annuel de la Chine.

## Resumen

**Objetivo:**

Estimar el coste económico de la enfermedad coronavirus-19 (COVID-19) en 31 regiones administrativas a nivel provincial y en su totalidad en China.

**Métodos:**

Utilizamos los datos de los informes del gobierno, las guías clínicas y otras publicaciones para estimar los principales componentes del coste de la COVID-19 desde el 1 de enero al 31 de marzo de 2020. Estos componentes fueron: identificación y diagnóstico de contactos cercanos; casos sospechosos y casos confirmados de COVID-19; tratamiento de los casos de COVID-19; cuarentena obligatoria de contactos cercanos y casos sospechosos; y pérdidas de productividad para todos los residentes afectados. El principal resultado fue los costes totales de la atención sanitaria y de la sociedad.

**Resultados:**

El total estimado de los costes de atención sanitaria y de la sociedad asociados con la COVID-19 fue de 4.260 millones de yuanes chinos (¥; 0,62 mil millones de dólares estadounidenses) y 2.646,70 mil millones de yuanes (383,02 mil millones de dólares estadounidenses), respectivamente. La atención hospitalaria representó el 44,2% (0,95 mil millones/2,15 mil millones de yenes) de los costes de la atención sanitaria rutinaria, seguida de los medicamentos, que representaron el 32,5% (0,70 mil millones/2,15 mil millones de yenes). Las pérdidas de productividad representaron el 99,8% (2.641,61 mil millones/2.646,70 mil millones de yenes) de los costes de la sociedad, que se atribuyeron principalmente al efecto de las políticas de restricción de movimientos en las personas que no tenían COVID-19. Los costes sociales fueron más sensibles a los costes salariales y al número de días de trabajo perdidos debido a las políticas de restricción de movimiento. La provincia de Hubei tenía el mayor coste de atención sanitaria, mientras que la provincia de Guangdong tenía el mayor coste social.

**Conclusión:**

Nuestros resultados destacan la alta carga económica del brote de COVID-19 en China. Las medidas de control para evitar la propagación de la enfermedad dieron lugar a costes sustanciales por pérdidas de productividad que ascendieron al 2,7% (382,29 mil millones de dólares estadounidenses/14,14 millones de millones de dólares estadounidenses) del producto interno bruto anual de China.

## ملخص

الغرض تقدير التكلفة الاقتصادية بسبب مرض فيروس كورونا 19 (كوفيد 19) في 31 منطقة إدارية على مستوى المقاطعات، وفي الصين بشكل إجمالي.

الطريقة قمنا باستخدام بيانات من التقارير الحكومية، والإرشادات الإكلينيكية، وغيرها من المطبوعات لتقدير مكونات التكلفة الرئيسية لكوفيد 19، خلال الفترة من 1 يناير/كانون ثاني إلى 31 مارس/آذار 2020. وكانت هذه المكونات هي: تحديد المخالطين عن قرب وتشخيص حالاتهم؛ والحالات المشتبه فيها والحالات المؤكدة للإصابة بكوفيد 19؛ وعلاج حالات كوفيد 19؛ والحجر الصحي الإجباري للمخالطين عن قرب والحالات المشتبه فيها؛ وخسائر الإنتاجية لجميع السكان المتضررين. كانت النتائج الأولية هي إجمالي تكاليف الرعاية الصحية والمجتمعية.

النتائج بلغ الإجمالي التقديري لتكاليف الرعاية الصحية والمجتمعية لمرض كوفيد 19، مبلغ 4.62 مليار يوان صيني (0.62 مليار دولار أمريكي)، و2646.70 مليار يوان (383.02 مليار دولار أمريكي)، على التوالي. شكلت رعاية المرضى بالمستشفيات 44.2% (0.95 مليار يوان/2.15 مليار يوان) من تكاليف الرعاية الصحية الروتينية، تليها الأدوية والتي تمثل 32.5% (0.70 مليار يوان/2.15 مليار يوان). شكلت خسائر الإنتاجية 99.8% (2641.61 مليار يوان/2646.70 مليار يوان) من التكاليف المجتمعية، والتي تُعزى في الغالب إلى تأثير سياسات تقييد الحركة على الأشخاص الذين لم يتعرضوا للإصابة بكوفيد 19. كانت التكاليف المجتمعية أكثر حساسية لتكاليف الرواتب وعدد أيام العمل الضائعة بسبب سياسات تقييد الحركة. سجلت مقاطعة هوبي أعلى تكلفة للرعاية الصحية، بينما سجلت مقاطعة جوانجدونج أعلى تكلفة مجتمعية.

الاستنتاج تركز النتائج لدينا على العبء الاقتصادي المرتفع لتفشي مرض كوفيد 19 في الصين. أدت تدابير المكافحة لمنع انتشار المرض، إلى تكاليف كبيرة من خسائر الإنتاجية بلغت 2.7% (382.29 مليار دولار أمريكي/14.14 تريليون دولار أمريكي) من الناتج المحلي الإجمالي السنوي للصين. ****

## 摘要

**目的:**

旨在估算新型冠状病毒肺炎（新冠肺炎）对中国 31 个省级行政区域造成的经济成本, 包括卫生系统成本和社会成本。

**方法:**

我们采用政府报告、临床指南和其他出版物中所汇报数据，估算了 2020 年 1 月 1 日至 3 月 31 日期间新冠肺炎的相关成本。成本构成包括：密切接触者的识别和诊断；新冠肺炎疑似病例和确诊病例的识别和诊断 ；新冠肺炎病例的治疗 ；密切接触者和疑似病例的强制隔离 ；以及所有受影响居民的生产力损失。

**结果:**

新冠肺炎相关卫生和社会成本总额估算值分别 为 42.6 亿元人民币（6.2 亿美元）和 26,467.0 亿元人民 币（3,830.2 亿美元）。住院治疗费用占常规医疗费用 的 44.2%（9.5 亿元人民币 / 21.5 亿元人民币），其次是 药品费用，占 32.5%（7.0 亿元人民币 / 21.5 亿元人民币）。 生产力损失占社会成本的 99.8%（26,416.1 亿元人民币/ 26,467.0 亿元人民币），这主要是因行动限制政策对未 感染新冠肺炎的人们造成影响所致。社会成本对因行动限制政策而损失的工资成本和工作天数最为敏感。 湖北省的医疗成本最高，而广东省的社会成本最高

**结论:**

我们的研究结果表明，新冠肺炎疫情对中国 造成了非常沉重的经济负担。为防止疾病传播而采取 的控制措施导致生产力大幅损失，相当于中国 年度国内生产总值的 2.7%（3,822.9 亿美元 / 14.14 万 亿美元）。

## Резюме

**Цель:**

Оценить экономический ущерб, нанесенный коронавирусной инфекцией (COVID-19) в 31 административном районе на уровне провинции и в целом по Китаю.

**Методы:**

Авторы использовали данные отчетов правительства, клинических рекомендаций и других публикаций для оценки основных компонентов затрат, связанных с COVID-19, в период с 1 января по 31 марта 2020 года. Этими компонентами были следующие: идентификация и диагностика лиц, находившихся в тесном контакте с заболевшими; подозрение на COVID-19 и подтвержденные случаи заболевания; лечение выявленных случаев заболевания COVID-19; обязательный карантин лиц, находившихся в тесном контакте с заболевшими, и случаев с подозрением на наличие инфекции; потеря работоспособности затронутой части населения. Показателями основных результатов были общие расходы на здравоохранение и социальные издержки.

**Результаты:**

Общие оценочные расходы на здравоохранение и социальные издержки, связанные с COVID-19, составили 4,26 млрд китайских юаней (0,62 млрд долларов США) и 2646,70 млрд юаней (383,02 млрд долларов США) соответственно. На стационарное лечение приходилось 44,2% (0,95/2,15 млрд юаней) от планового уровня расходов на здравоохранение, далее следовали расходы на лекарственные препараты, составлявшие 32,5% (0,70/2,15 млрд юаней). На потерю работоспособности пришлось 99,8% (2641,61/2646,70 млрд юаней) социальных издержек, которые в основном были связаны с влиянием введенных ограничений на передвижения людей, которые не болели COVID-19. Издержки для общества были наиболее чувствительны к расходам на заработную плату и количеству потерянных рабочих дней по причине ограничения передвижения. В провинции Хубэй наблюдались самые высокие расходы на здравоохранение, в то время как в провинции Гуандун отмечались самые высокие социальные издержки.

**Вывод:**

Полученные результаты подчеркивают высокое экономическое бремя вспышки COVID-19 в Китае. Меры контроля по предотвращению распространения заболевания привели к значительным расходам в результате потери работоспособности, составившей 2,7% (382,29 млрд / 14,14 трлн долларов США) годового валового внутреннего продукта Китая.

## Introduction

Coronavirus disease 2019 (COVID-19) is an infectious disease which results in substantial morbidity and mortality in some population groups. By September 2020, over 32.7 million cases of COVID-19 had been confirmed worldwide, of which 90 966 were in China.^1^

Prevention and treatment of COVID-19 can be expensive. According to Chinese clinical guidelines,^2^^,^^3^ all confirmed cases of COVID-19 should receive inpatient care. Moreover, patients with critical COVID-19 often require costly treatment such as mechanical ventilation and extracorporeal membrane oxygenation, potentially substantially increasing health-care costs. The societal cost of COVID-19 could be even greater. To prevent disease transmission, a series of emergency measures were implemented by the Chinese government,^4^ including isolation of COVID-19 cases, 14-day quarantine for close contacts of COVID-19 cases, lockdown of Wuhan city and adjacent areas, travel restrictions and extension of the Chinese New Year holiday period. While these containment strategies successfully reduced the transmission of COVID-19,^5^ they inevitably caused a considerable loss in productivity.

This study assessed the health and societal costs of the COVID-19 outbreak in 31 provincial-level administrative regions in mainland China.

## Methods

We conducted and reported our study according to the cost-of-illness checklist.^6^

### Study population

**Fig. 1:**
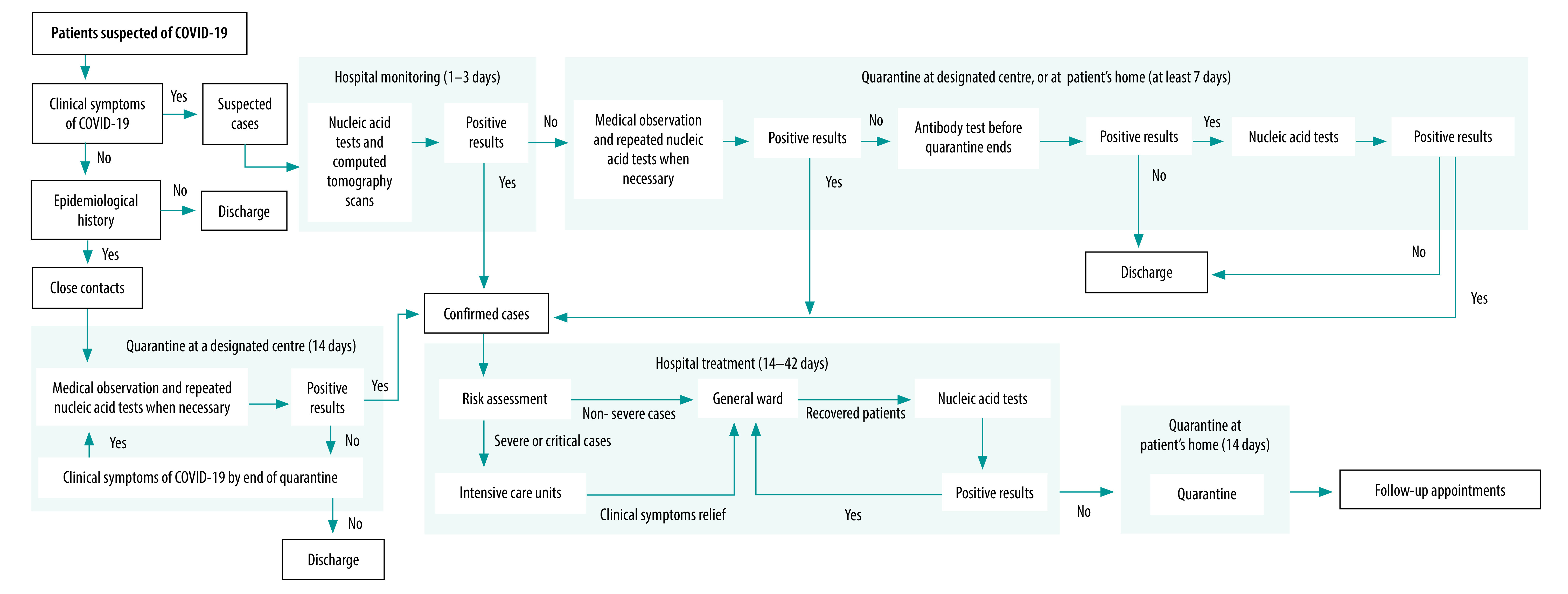
Simplified diagnostic and treatment pathway for COVID-19, China, 2020

The population of interest was all residents in mainland China, which has 31 provincial-level administrative regions – 22 provinces, five autonomous regions (Guangxi Zhuang, Inner Mongolia, Ningxia Hui, Tibet and Xinjiang Uyghur) and four municipalities (Beijing, Chongqing, Shanghai and Tianjin). We divided the population into four mutually exclusive patient subgroups, based on their experience of COVID-19: (i) asymptomatic close contacts of suspected or confirmed cases of COVID-19, who were eventually diagnosed as COVID-19 negative; (ii) symptomatic suspected cases with or without close contact history with existing suspected or confirmed cases, who were eventually diagnosed as COVID-19 negative; (iii) confirmed cases of COVID-19, including those previously assessed as close contacts or suspected cases; and (iv) people not considered to have been exposed to COVID-19. We further divided confirmed cases into non-severe, severe and critical COVID-19, according to the disease severity (Box 1). Fig. 1 shows the diagnostic and treatment pathway for each patient subgroup; also described in the data repository.^8^

Box 1 **Definition of close contacts, suspected cases and confirmed cases of COVID-19, China, 2020**

***Close contact***

An asymptomatic person who has had close (less than 1 m), unprotected (without personal protective equipment) contact with suspected cases or confirmed cases (see definitions below), 2 or fewer days before the onset of their symptoms.

***Suspected case***

A person who has one epidemiological history criteria and meets two clinical symptoms criteria, or has no epidemiological history but meets all three clinical symptoms criteria.

• Epidemiological history. Fourteen days before the onset of the disease, the person has: (i) travelled to or lived in a high-risk region or country; or (ii) had direct contact with confirmed cases (definition below); or (iii) had direct contact with someone with a fever or respiratory symptoms in a high-risk region or country; or (iv) been to a place with disease clustering – defined as two or more cases with fever and/or respiratory symptoms occurring at places such as homes, offices and school classrooms.

• Clinical symptoms. The person has: (i) a fever and/or respiratory symptoms; (ii) the following imaging features of COVID-19 after computerized tomography of their chest – multiple patchy shadows and interstitial changes, particularly at the periphery of the lungs, multiple ground-glass opacities and infiltrates in both lungs, or in severe cases, lung consolidation and pleural effusion; (iii) normal or decreased white blood cell count in the early stage of the disease, or normal or decreased lymphocyte count over time.

***Confirmed case***

A suspected case that meets one of the following criteria: (i) positive result of the nucleic acid test for SARS-CoV-2; (ii) DNA sequencing results indicating high sequence similarity to known SARS-CoV-2 sequences; (iii) positive result for the serum-specific antibodies (IgM and IgG) of COVID-19.

Severity of disease in confirmed cases is categorized as follows.

• Non-severe cases: mild cases (mild clinical symptoms with no signs of pneumonia on imaging) and moderate cases (symptoms such as fever and respiratory tract symptoms, and signs of pneumonia on imaging).

• Severe cases: presence of any of the following conditions: (i) respiratory rate ≥ 30 breaths/min; (ii) oxygen saturation ≤ 93.0% in a resting state; (iii) ratio of partial pressure arterial oxygen and the fraction of inspired oxygen ≤ 300 mm Hg.

• Critical cases: presence of any of the following conditions: (i) respiratory failure requiring mechanical ventilation; (ii) shock; (iii) other organ failure that requires monitoring and treatment in an intensive care unit.

COVID-19: coronavirus disease 2019; DNA: deoxyribonucleic acid; Ig: immunoglobulin; SARS-CoV-2: severe acute respiratory syndrome coronavirus 2. Note: Definitions are based on Chinese guidelines.^2^^,^^3^^,^^7^

COVID-19: coronavirus disease 2019.

Notes: The pathway is based on national and local clinical guidelines.^4^^,^^5^ Definitions of clinical symptoms and epidemiological history are in the data repository.^8^ Positive results for people monitored in hospital were positive nucleic acid test results and/or presence of lung imaging features consistent with coronavirus pneumonia.

### Outcomes

We estimated direct health-care costs, direct non-health-care cost and productivity losses for each region and for mainland China as a whole (Box 2). We calculated all costs in Chinese yuan (¥) at the 2019 value and converted to United States dollars (US$) using the annual exchange rate for 2019: US$ 1.00 = ¥ 6.91.^9^

Box 2 **Components of the cost categories used in the COVID-19 costing study, China, 2020**

**Direct health-care costs**

Routine health care: identification, diagnosis, treatment and follow-up of people with suspected or confirmed COVID-19.

Non-routine health care: (i) risk subsidy for front-line health professionals who work with suspected and/or confirmed cases; and (ii) emergency funds for construction of temporary emergency buildings (i.e. Huoshenshan and Leishenshan hospitals, and Wuhan mobile cabin hospital), and non-routine procurement of additional medical supplies and equipment (e.g. personal protective equipment).

**Direct non-health-care costs**

Compulsory quarantine for close contacts and suspected cases. The quarantine cost can be covered by the local government, or by the quarantined individual, or jointly, depending on local policies.

**Productivity losses**

These losses include: (i) employed close contacts, suspected cases or confirmed cases who lost work time due to their quarantine and/or inpatient care; and (ii) any employed individuals who lost work time due to government policies controlling population movement (these individuals include people not considered to have had COVID-19).

COVID-19: coronavirus disease 2019

### Study period

Although COVID-19 was first identified in China in December 2019, 99.96% (74 648/74 675) of confirmed cases were identified in January and February 2020.^10^ From 6 March 2020, the number of new cases a day fell below 100, and no new cases were identified in 29 regions. Therefore, we calculated costs for the period from 1 January to 31 March 2020.

### Cost estimation

There are two approaches to estimate the cost of illness: the bottom-up approach and the top-down approach.^6^ The bottom-up approach multiplies the average cost of the illness per patient by the prevalence of the illness. The top-down approach uses aggregated data and a population-attributable fraction to assign a percentage of total expenditure to the disease of interest. Because published total expenditure on COVID-19 was lacking (details in the data repository),^8^ we used the bottom-up approach. We estimated unit costs, *p_x_*, at the patient or individual level for each component, *x*, of the overall burden of disease. We calculated the overall cost, *C*, as:
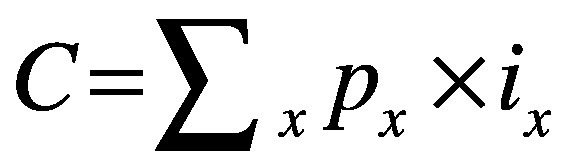
(1)where *i_x_* is the number of individuals affected.

#### Epidemiological data

Over the period of interest, the National Health Commission of the People’s Republic of China published national data on COVID-19 daily.^11^ However, detailed regional information was only published for Hubei province. Therefore, we manually extracted the number of newly identified close contacts, suspected cases and confirmed cases in each region from the daily updates reported by the local health commission of each region (details in the data repository).^8^ While all regions reported complete data for the number of confirmed cases and the numbers of deaths of confirmed cases, data were incomplete for the number of close contacts and/or suspected cases. We estimated these missing data either from published reports, or from the reported regional number of confirmed cases,^11^ assuming the same ratio between the number of close contacts or suspected cases and confirmed cases across regions. 

#### Direct health-care cost

We used information in the published literature^12^^,^^13^ and clinical guidelines,^2^^,^^3^ supplemented with expert opinion where necessary, to estimate the health-care resources used for close contacts, suspected cases and confirmed cases. Shanghai is one of the few regions in China which reports full unit cost data.^14^ To calculate the unit costs for other regions, we calculated a health-care industry salary index (details in the data repository).^8^ We calculated a weight (*w_r_*) for each region as:
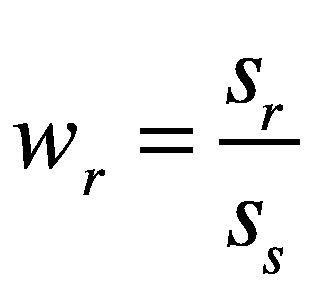
(2)where *s_r_* is the ratio of the average health-care industry salary in the region and *s_s_* is the average health-care industry salary in Shanghai.^15^ We then estimated regional unit costs (*p_r_*) as:
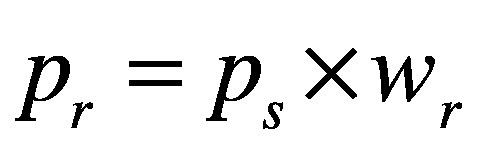
(3)where *p_s_* is the unit costs derived from Shanghai.^14^

According to the State Council, 42 600 front-line health professionals worked with suspected and/or confirmed COVID-19 cases.^16^ The daily risk subsidy for front-line health professionals was estimated to be ¥ 300.00 per person.^17^ We estimated the emergency funds (for construction of temporary emergency buildings and non-routine procurement of additional medical supplies and equipment) based on the budget plans of the Ministry of Finance and the National Development and Reform Commission (data repository).^8^ For reusable equipment, we only included the cost attributable to the 3-month period of the study in our analysis. Calculations and results for emergency funds are in the data repository.^8^

#### Direct non-health-care cost

We estimated a daily cost of quarantine in Shanghai to be ¥ 75.00 (US$ 10.85), assuming that 50.0% of people quarantined at home at zero cost and 50.0% quarantined at a designated centre at the cost of ¥ 150.00 (US$ 21.71) a day. We calculated the regional quarantine costs per person (*QC_re_*) per person by category of exposure (*e*), as:
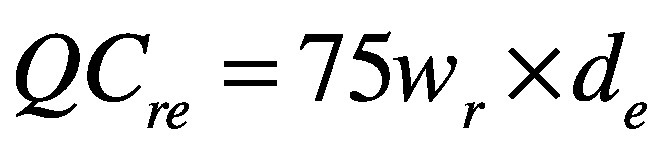
(4)where *w_r_* is the regional weight described earlier and *d_e_* is the estimated duration of quarantine.

The average cost of quarantine for close contacts and suspected cases was ¥ 1246.00 (US$ 180.32) and ¥ 735.00 (US$ 106.37) per person, respectively. We calculated the overall cost of quarantine (*TQC*) as:
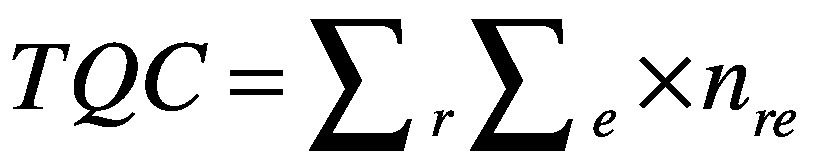
(5)where *n_re_* is the number of people quarantined by region (*r*) and exposure (*e*) category. Details on methods and results are in the data repository.^8^

#### Loss in productivity

We used the human capital approach to estimate productivity losses. For people not considered to have been exposed to COVID-19, we calculated costs by region (*CP_r_*) as:
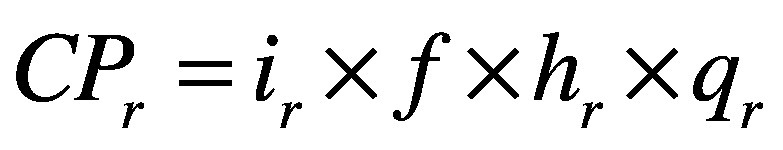
(6)where *i_r_* is the mean daily wage rate by region, *f* is the proportion of the population in employment, *h_r_* is the mean number of days lost by region, and *q_r_* is the regional population.

We obtained regional employment statistics from the China Statistical Yearbook 2019.^15^ The national average daily wage was ¥ 271.94 (US$ 39.35), ranging from ¥ 204.67 (US$ 29.62) in Heilongjiang province to ¥ 486.43 (US$ 70.40) in Beijing. The national unemployment rate was 3.0%, ranging from 1.4% in Beijing to 4.0% in Heilongjiang province. Data were not available on the employment status for close contacts, suspected cases and confirmed cases. Therefore, we estimated the employment rate, *f*, for each patient subgroup at 54.0% based on the age and sex distribution of confirmed cases, the legal working age (16 years) and official retirement age (60 years for men and 50 years for women), and the national unemployment rate (3.0%). Employment rate calculations are in the data repository.^8^ We estimated the average number of working days lost due to restrictions on movement for people not considered to have contracted COVID-19 as 23.26 days, based on the Baidu migration index,^18^^,^^19^ which tracks the proportion of workers returning from their hometowns to work after the Chinese New Year holiday. Close contacts, suspected cases and confirmed cases may have experienced more working days lost due to their quarantine and/or hospitalization.^12^^,^^13^^,^^20^ Working days lost for these people depended on the start and end date of their quarantine and/or hospitalization, and whether these dates overlapped with the extended Chinese New Year holiday and the study period. We limited productivity losses from COVID-19 deaths to the study period in the base case analysis. Calculations of the working days lost for each patient subgroup are in the data repository.^8^

### Sensitivity analysis

To determine which parameters were key cost drivers we conducted a sensitivity analysis. We identified costs that contributed to 10.0% or more of the total health-care costs and societal costs and varied the parameters for use of resources and unit cost. We used available data or our judgement to inform the ranges for the selected parameters.

## Results

During the study period, there were 707 913 close contacts, 126 032 suspected cases and 81 879 confirmed cases in mainland China (Table 1). Of confirmed cases, 83.2% (68 127/81 879) were from Hubei province. Of close contacts and suspected cases, 5.2% (36 598/707 913) and 65.0% (81 879/126 032), respectively, were diagnosed with COVID-19. As regards severity, 81.5% (66 732/81 879) of the confirmed COVID-19 cases were non-severe, 13.8% (11 299/81 879) were severe and 4.7% (3848/81 879) were critical. Details by region are in the data repository.^8^

**Table 1:** Close contacts, suspected cases and confirmed cases of COVID-19 by region, China, January–March 2020

Region	No. of close contacts				No. of suspected cases				No. of confirmed cases		
	Total	Diagnosis			Total	Diagnosis			Total	Survival outcome	
		Non-COVID-19	COVID-19			Non-COVID-19	COVID-19			Survived	Died
Anhui province	28 981	*27 445*	*1 536*		1 129	*139*	*990*		990	984	6
Beijing	4 164	*3 943*	*221*		*2 905*	*2 325*	*580*		580	572	8
Chongqing	23 803	*22 542*	*1 261*		*2 900*	*2 321*	*579*		579	573	6
Fujian province	13 315	*12 609*	*706*		609	*264*	*345*		345	344	1
Gansu province	4 337	*4 107*	*230*		*691*	*553*	*138*		138	136	2
Guangdong province	*41 136*	*38 956*	*2 180*		*7 517*	*6 016*	*1 501*		1 501	1 493	8
Guangxi Zhuang autonomous region	16 216	*15 357*	*859*		816	*562*	*254*		254	254	0
Guizhou province	2 577	2 508	69		*736*	*589*	*147*		147	145	2
Hainan province	6 574	*6 226*	*348*		*841*	*673*	*168*		168	162	6
Hebei province	11 143	10 622	521		690	*367*	*323*		323	317	6
Heilongjiang province	16 619	16 491	128		1 535	*1 051*	*484*		484	471	13
Henan province	40 019	*37 898*	*2 121*		*6 390*	*5 114*	*1 276*		1 276	1 254	22
Hubei province	278 179	*263 437*	*14 742*		68 127^a^	*0*	*68 127^b^*		*68 127^b^*	64 609	*4 483^b^*
Hunan province	27 331	*25 883*	*1 448*		*5 098*	*4 080*	*1 018*		1 018	1 014	4
Inner Mongolia autonomous region	3 123	*2 957*	*166*		111^a^	*0*	*111*		111	110	1
Jiangsu province	12 843	*12 162*	*681*		*3 235*	*2 589*	*646*		646	646	0
Jiangxi province	27 310	*25 863*	*1 447*		*4 693*	*3 756*	*937*		937	936	1
Jilin province	3 994	*3 782*	*212*		418	*320*	*98*		98	97	1
Liaoning province	3 729	*3 531*	*198*		*701*	*561*	*140*		140	139	1
Ningxia Hui autonomous region	4 719	*4 469*	*250*		75^a^	*0*	*75*		75	75	0
Qinghai province	437	*414*	*23*		18^a^	*0*	*18*		18	18	0
Shaanxi province	20 011	*18 951*	*1 060*		1 025	*770*	*255*		255	252	3
Shandong province	20 733	*19 634*	*1 099*		*3 876*	*3 102*	*774*		774	767	7
Shanghai	*14 142*	*13 393*	*749*		*2 584*	*2 068*	*516*		516	510	6
Shanxi province	4 564	4 350	214		301	*164*	*137*		137	137	0
Sichuan province	*15 128*	*14 326*	*802*		552^a^	*0*	*552*		552	549	3
Tianjin	3 008	*2 849*	*159*		*871*	*697*	*174*		174	171	3
Tibet autonomous region	32	*30*	*2*		1	*0*	*1*		1	1	0
Xinjiang Uyghur autonomous region	*2 083*	*1 973*	*110*		*381*	*305*	*76*		76	73	3
Yunnan province	10 899	*10 321*	*578*		*911*	*729*	*182*		182	180	2
Zhejiang province	46 764	*44 286*	*2 478*		*6 295*	*5 038*	*1 257*		1 257	1 256	1
**Total of all regions**	**707 913**	**671 315**	**36 598**		**126 032**	**44 153**	**81 879**		**81 879**	**77 280**	**4 599**
**Total^c^**	**NR**	**NR**	**NR**		***98 200*^b^**	**NR**	**NR**		***81 879*^b^**	**NR**	***4 602*^b^**

COVID-19: coronavirus disease 2019; NR: not reported.

^a^ The original number of suspected cases reported was lower than the number of confirmed cases. In such cases, the number of suspected cases was corrected to the number of confirmed cases (as a conservative proxy).

^b^ Adjusted based on the corrected number reported by the Wuhan government on 17 April 2020.^21^

^c^ Reported by the National Health Commission of the People’s Republic of China.^11^

Note: We obtained the data in regular font from the local health commission of each region. We calculated or estimated, or corrected the data in italic font.

Table 2 shows the health-care cost per person for each patient subgroup, based on the estimated use of resources and the unit costs from Shanghai.^15^ The health-care cost of managing close contacts and suspected cases diagnosed as COVID-19 negative was ¥ 584.08 (US$ 84.53) and ¥ 973.70 **(**US$ 140.91) per person, respectively. The weighted average cost of treating a confirmed case of COVID-19 was ¥ 22 061.94 (US$ 3192.76), ranging from ¥ 6488.90 (US$ 939.06) for non-severe cases to ¥ 176 744.05 (US$ 25 578.01) for critical cases (data repository).^8^

**Table 2:** Health-care costs for close contacts, suspected cases and confirmed cases of COVID-19, China, January–March 2020

Cost component	Probability of using services	National unit cost, ¥	Resource use	Cost per person, ¥
**Close contact diagnosed as COVID-19 negative**				
Case identification	1.00	15.68 per case	1	15.68
Nucleic acid test	1.00	70.00 per test	2	140.00
Medical observation	1.00	35.00 per day	12.24	428.40
**Total**				**584.08 (US$ 84.53)**
**Suspected case diagnosed as COVID-19 negative**				
Outpatient consultation	1.00	12.60 per consultation	1	12.60
Nucleic acid test	1.00	70.00 per test	2	140.00
Other laboratory tests	1.00	282.10 per test	1	282.10
Computed tomography scan	1.00	140.00 per scan	1	140.00
Hospital bed days	1.00	77.00 per day	2	154.00
Medical observation	1.00	35.00 per day	7	245.00
**Total**				**973.70 (US$ 140.91)**
**Confirmed case, non-severe**				
Identification and diagnosis^a^	0.45	636.30 per case	1	286.34
Identification and diagnosis^b^	0.55	549.92 per case	1	302.46
Inpatient care^c^	1.00	389.40 per day	14	5 451.64
Medicines^d^	1.00	27.50 per day	14	385.04
Treatment for pre-existing conditions	0.26	10.50 per day	14	38.22
Follow-up appointment	1.00	12.60 per appointment	2	25.20
**Total**				**6** **488.90 (US$ 939.06)**
**Confirmed case, severe**				
Identification and diagnosis^a^	0.45	636.30 per case	1	286.34
Identification and diagnosis^b^	0.55	549.92 per case	1	302.46
Inpatient care^c^	1.00	592.79 per day	28	16 598.11
Medicines^d^	1.00	1394.95 per day	28	39 058.64
Treatment for pre-existing conditions	0.26	645.25 per day	28	4 697.44
Oxygen therapy	1.00	3.42 per hour	112	383.38
Follow-up appointment	1.00	12.60 per appointment	2	25.20
**Total**				**61 351.57 (US$ 8 878.66)**
**Confirmed case, critical**				
Identification and diagnosis^a^	0.45	636.30 per case	1	286.34
Identification and diagnosis^b^	0.55	549.92 per case	1	302.46
Inpatient care^c^	1.00	771.58 per day	42	32 406.43
Medicines^d^	1.00	1 628.43 per day	42	68 394.24
Treatment for pre-existing conditions	0.26	481.03 per day	42	5 252.80
Tracheostomy and tracheal intubation	1.00	175.00 each	1	175.00
Use of ventilator (including muscle relaxants)	0.71	Day 1: 1 892.38; Day 2 onwards: 1 402.38	30	30 218.58
Extracorporeal membrane oxygenation	0.12	Day 1: 42 000.00; Day 2 onwards: 7 000.00	20	21 000.00
Artificial kidney	0.17	Day 1: 5 600.00; Day 2 onwards: 4 200.00	20	14 518.00
Plasma exchange	0.17	4 900.00 per exchange	5	4 165.00
Follow-up appointment	1.00	12.60 per appointment	2	25.20
**Total**				**176 744.05 (US$ 25 578.01)**

COVID-19: coronavirus disease 2019; ¥: Chinese yuan; US$: United States dollars.

^a^ Identified from close contacts.

^b^ Identified from suspected cases.

^c^ Hospital bed days, nursing, blood gas analyses and laboratory tests.

^d^ Anti-infective medicines and nutrition support.

Notes: Details for each individual cost component are in the data repository.^8^ For the confirmed cases, we assumed that 45% of them were identified from close contacts, whilst 55% were identified from suspected cases. Therefore, the cost of identification and diagnosis for all confirmed cases was calculated as the multiplication of the cost per case and 0.45 for close contacts and 0.55 for suspected cases.

**Fig. 2:**
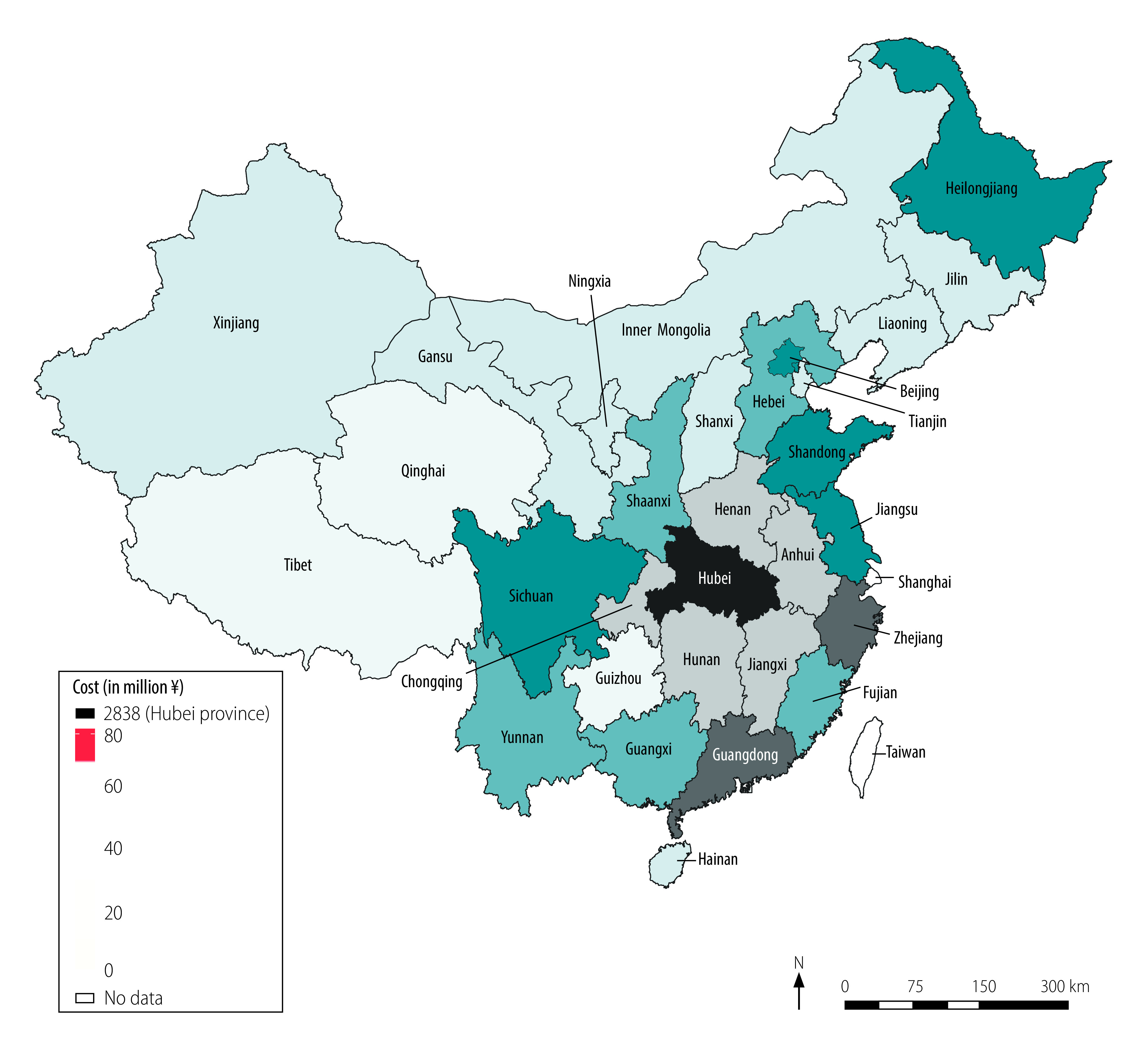
Health-care cost of COVID-19 by region, China, January–March 2020

**Fig. 3:**
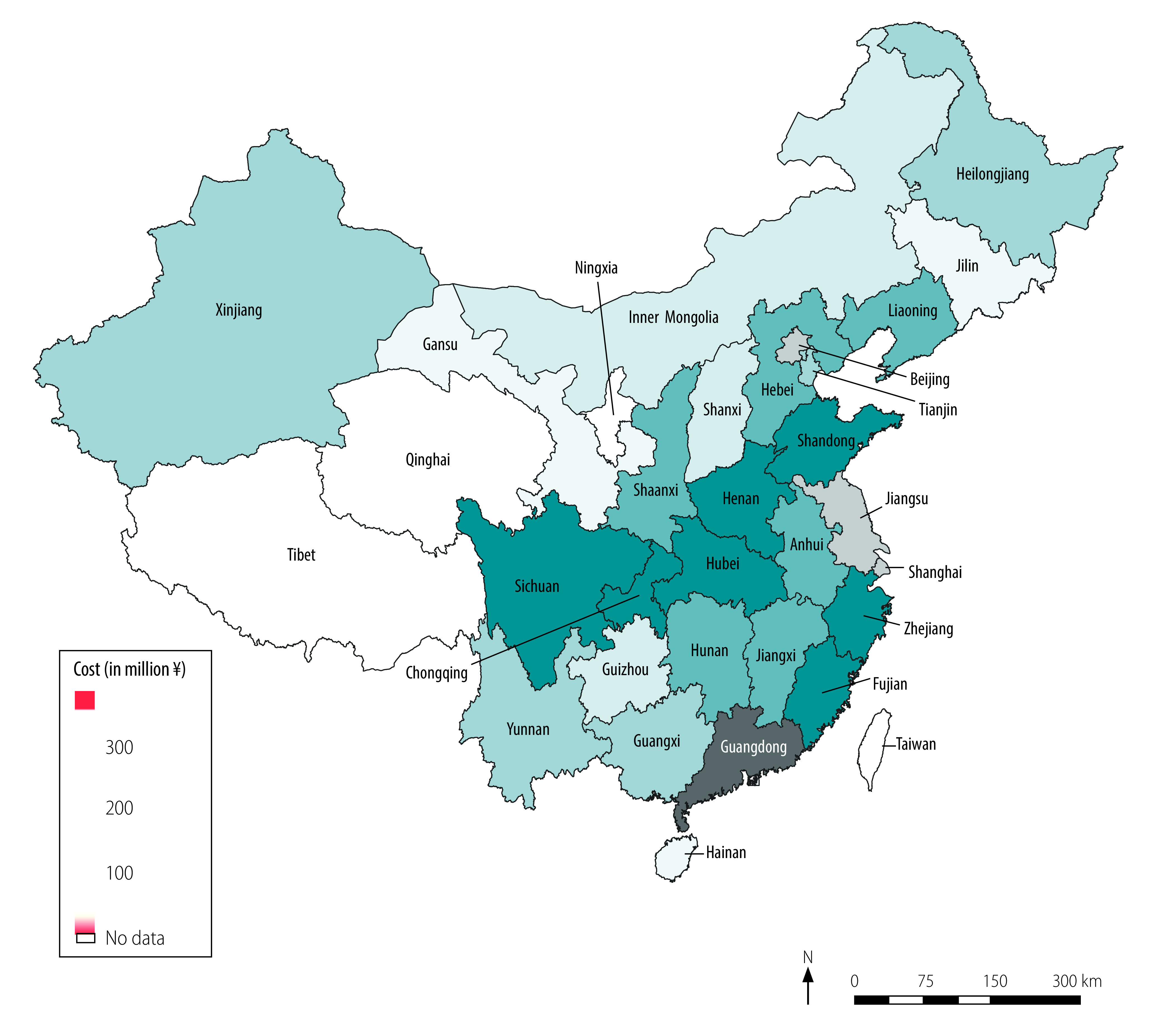
Societal cost of COVID-19 by region, China, January–March 2020

We calculated costs of routine health-care services, quarantine and productivity losses, and total health-care and societal costs (Table 3). We estimated routine health-care costs at ¥ 2.15 (US$ 0.31) billion. Inpatient care accounted for 44.2% (¥ 0.95 billion/¥ 2.15 billion) of routine health-care costs, followed by medicines, which accounted for 32.5% (¥ 0.70 billion/¥ 2.15 billion), and medical observation of close contacts and suspected cases, which accounted for 13.0% (¥ 0.28 billion/¥ 2.15 billion). Confirmed cases who died accounted for 32.4% (¥ 0.70 billion/¥ 2.15 billion) of routine health-care costs, severe cases who survived accounted for 27.8% (¥ 0.60 billion/¥ 2.15 billion), and non-severe confirmed cases who survived accounted for 17.4% (¥ 0.37 billion/¥ 2.15 billion). We estimated the cost of quarantine at ¥ 0.84 billion (US$ 0.12 billion), 96.0% (¥ 0.80 billion/¥ 0.84 billion) of which was spent on close contacts diagnosed as COVID-19 negative (Table 3). Our estimation of productivity losses was ¥ 2641.61 billion (US$ 382.29 billion), 99.9% (¥ 2638.38 billion/¥ 2641.61 billion) of which were attributable to lost working time as a result of movement restriction policies for people not considered to have had COVID-19 (Table 3). The total societal cost of COVID-19 was ¥ 2646.70 billion (US$ 383.02 billion; Table 3), which is equivalent to 2.7% of China’s gross domestic product (GDP) in 2019 (US$ 14.14 trillion).^22^ Health-care costs accounted for only 0.2% (¥ 4.26 billion/¥ 2646.70 billion) of the societal cost while productivity losses accounted for 99.8% (¥ 2641.61 billion/¥ 2646.70 billion). Fig. 2 and Fig. 3 show the health-care cost and societal cost for each region, respectively. The health-care cost for Hubei province alone accounted for 66.7% (¥ 2.84 billion/¥ 4.26 billion) of the national health-care cost (Fig. 2). Guangdong province incurred the highest societal cost, followed by Jiangsu province and Beijing (Fig. 3).

**Table 3:** Cost of COVID-19 according to cost component and COVID-19 diagnosis, China, January–March 2020

Cost component	Cost, million ¥										Total cost, million ¥ (million US$)
	People not considered to have had COVID-19	Close contacts diagnosed as COVID-19 negative	Suspected cases diagnosed as COVID-19 negative		Confirmed cases surviving				Confirmed cases died		
					Non-severe	Severe	Critical				
**Routine health care**											
Identification and diagnosis	N/A	95.16	13.30		33.91	5.74	0.27		2.32		150.70 (21.81)
Medical observation^a^	N/A	261.85	17.50		N/A	N/A	N/A		N/A		279.35 (40.43)
Inpatient care	N/A	N/A	13.46		314.00	169.70	46.79		405.31		949.26 (137.37)
Medicines	N/A	N/A	N/A		22.18	377.07	30.92		267.86		698.03 (101.02)
Treatment for pre-existing conditions	N/A	N/A	N/A		2.20	45.43	2.37		20.54		70.54 (10.21)
Follow-up for recovered cases	N/A	N/A	N/A		1.45	0.25	0.01		N/A		1.71 (0.25)
**Subtotal**	N/A	357.01	44.26		373.74	598.19	80.36		696.03		2 149.59 (311.08)
**Non-routine health care^b^**	N/A	N/A	N/A		N/A	N/A	N/A		N/A		2 106.81 (304.89)
**Quarantine for test-negative cases**	N/A	803.17	33.41		N/A	N/A	N/A		N/A		836.58 (121.07)
**Productivity loss**	2 638 379.36	2 635.33	169.86		328.63	62.10	3.41		26.51		2 641 605.20 (382 287.29)
**Total (societal cost)**	2 638 379.36	3 795.51	247.53		702.37	660.29	83.77		722.54		2 646 698.18 (383 024.34)^c^

COVID-19: coronavirus disease 2019; N/A: not applicable; US$: United States dollars;

¥: Chinese yuan.

^a^ Medical observation of close contacts and/or suspected cases before receiving a diagnosis of COVID-19.

^b^ Includes risk subsidy for health-care staff and emergency funds for construction of temporary emergency buildings and non-routine procurement of additional medical supplies and equipment.

^c^ Column total. We could not assign the cost of non-routine health care to any specific individual patient group so we only report the total cost of non-routine health care in the last column; therefore, the cost of non-routine health care is not reflected in the total societal cost for each patient subgroup (last row).

COVID-19: coronavirus disease 2019; ¥: yuan.

COVID-19: coronavirus disease 2019; ¥: yuan.

The results of the sensitivity analyses are reported in the data repository.^8^ The direct health-care cost was most sensitive to the proportion of confirmed cases with severe or critical disease, and the health-care cost per person for treating severe and critical cases. The cost of the loss in productivity was most sensitive to the number of working days lost for people not considered to have had COVID-19, the national average daily salary, and assumptions on the effect of movement restriction policies on worker productivity.

## Discussion

We estimated the health-care and societal costs associated with the COVID-19 outbreak in China for the first 3 months of 2020 to be ¥ 4.26 billion (US$ 0.62 billion) and ¥ 2646.70 billion (US$ 383.03 billion), respectively. Although the health-care cost per person for confirmed cases was high, 99.9% of the societal cost was attributable to productivity losses in people not considered to have had COVID-19. These findings reflect the overall number of employed people in China (416.5 million), which is much larger than the number of confirmed cases (81 879 cases). Our estimated cost of productivity losses – ¥ 2641.61 billion (US$ 382.29 billion) – is comparable to the decrease in the Chinese GDP for the first quarter of 2020 compared with the same period in 2019: ¥ 1506.68 billion (US$ 218.04 billion).^22^

Hubei province, where most confirmed cases were identified, accounted for two thirds of the national health-care cost. The productivity loss was greatest for those regions with the highest number of employed people and/or the highest daily salary, such as Guangdong province (57.7 million employed people, ¥ 296.37, US$ 42.89, daily salary), Jiangsu province (42.2 million employed people, ¥ 279.41, US$ 40.44, daily salary) and Beijing (15.7 million employed people, ¥ 486.43, US$ 70.40, daily salary).

We did not identify any cost-of-illness studies for COVID-19 in our rapid review of the literature. Evidence on cost of illness is available for severe acute respiratory syndrome (SARS).^23^^–^^27^ To facilitate comparison of results, we inflated costs from the literature to 2019 values using a local consumer price index and converted to US$ using the annual exchange rate.^28^ Three studies^23^^–^^25^ reported the cost of managing patients with SARS; the health-care cost per case ranged from US$ 4151.00 in mainland China^24^ to US$ 362 700.00 in Canada.^23^ The cost for mainland China is similar to our estimate of US$ 3235 per COVID-19 case.^24^ An analysis of Chinese governmental health expenditure during 2002–2006 found that the SARS outbreak in 2003 increased governmental health expenditure by 4.1% (¥ 4.65 billion/¥ 113.39 billion).^29^ Another study used a simulation model to estimate the societal cost of SARS in 30 countries.^30^ The cost in mainland China was 1.03% (¥ 0.12 trillion/¥ 11.69 trillion) of GDP,^30^ which is comparable to our estimate of the societal cost of COVID-19 (2.7% of China’s GDP in 2019).^22^

The societal cost of COVID-19 is substantial and greatly outweighs the health-care cost. Our analysis, which demonstrates the effect of COVID-19 beyond the health-care system, justifies the redirection of resources from other sectors of the economy to strengthen health systems, as the potential productivity losses caused by a pandemic may far exceed the health-care cost. Despite a lack of evidence on their cost–effectiveness, unprecedented controls on people’s movements and ability to work have been imposed in several countries in an attempt to reduce the spread of COVID-19. Future work will examine the cost–effectiveness of these policies. Our data can help inform these analyses by providing the cost of identifying, diagnosing and treating patients with suspected or confirmed COVID-19. Our analysis underlines the importance of action to strengthen health systems, particularly the capacity to test for infection and trace contacts, which has been identified as one of the most cost-effective policy responses.^31^ Effective disease mitigation action will require international cooperation and considerable investment. Underinvestment in strengthening the capacity of health systems to tackle future pandemics could prove to be far costlier than the additional investment required.

Our study has several strengths. This study fills an important evidence gap by presenting the first cost-of-illness study of COVID-19. The study identified the cost of the COVID-19 pandemic in different sectors of the economy; such data are necessary to inform planning of services and the prioritization of research. Our data also provide important information for future economic evaluations of interventions for COVID-19. We accessed detailed data on use of resources in the 31 regions of mainland China, including incidence of close contacts, suspected cases and confirmed cases, from the local health commission of each region. We applied unit cost data adjusted to reflect relative price differences across provinces, and used clinician input from Shanghai and Hubei province to check the use of resources for each subgroup (close contacts, suspected and confirmed cases). We estimated productivity costs for close contacts, suspected cases and confirmed cases based on the duration of quarantine and/or treatment, and regional migration patterns after the end of the extended Chinese New Year holiday period.

Our analysis also has some limitations. First, we only covered the first 3 months of the epidemic and therefore could not capture the long-term economic effects of COVID-19. Future research is needed to assess the long-term economic impact of COVID-19 on the health-care system (e.g. for management of chronic diseases) and on society (e.g. reduced international trade and increased unemployment rates). Second, due to a lack of data, we could not include some cost components, such as productivity losses for carers of suspected and confirmed cases and out-of-pocket payments for travel to hospitals and over-the-counter medicines. Third, because of a shortage of nucleic acid tests in China in January 2020, not all patients suspected of having COVID-19 were tested.^3^ Therefore, the reported number of confirmed cases is likely to be an underestimate, especially in Hubei province. Fourth, our estimate of the number of working days lost, which we based on migration data, may have overestimated losses for people who worked from home. Fifth, we lacked some data on the incidence, demographic information and prognosis for close contacts and suspected cases, and had to estimate these data based on published literature and/or expert opinion. Finally, some positive effects of the restrictive measurements have been reported, such as reductions in crime rates,^32^ environmental improvements^33^ and a rapid increase in e-commerce.^34^ Analysis of the effects of these factors was beyond the scope of our study.

The results of our study highlight the substantial economic burden of the COVID-19 outbreak. Research is needed on the cost–effectiveness of different policies to control infectious diseases and developing capacity to limit the spread of disease while minimizing the impact on everyday life.
